# Genetic and contextual risks and developmental mechanisms underlying childhood aggression and young adult justice system involvement

**DOI:** 10.1017/S0954579426101898

**Published:** 2026-09-23

**Authors:** Savannah G. Ostner, Jenn-Yun Tein, Daniel S. Shaw, Erika Westling, Jazmin Brown-Iannuzzi, Kathryn Lemery-Chalfant

**Affiliations:** 1 Department of Psychology, Arizona State University, USA; 2 Department of Psychology, University of Pittsburgh, USA; 3 Oregon Research Institute, USA; 4 Department of Psychology, University of Virginia, USA

**Keywords:** aggression, antisocial behavior, developmental cascade, justice system involvement, polygenic risk score

## Abstract

Aggressive behavior, shaped by genetics and environmental or contextual influences, is expressed differently across development and may lead to criminal justice involvement through arrests and incarceration for some individuals. This study examined the differential impacts of early childhood contextual risk and polygenic risk on a developmental cascade from middle childhood physical aggression and dysregulation to adolescent antisocial behavior, to criminal justice system involvement by age 19, and whether these risks were mitigated by random assignment to the Family Check-Up (FCU) prevention program. The sample included high-risk toddler (*N* = 731; *M*_age_ = 28.2 months, *SD* = 3.28; 49.5% female, 50% White, 28% Black, 13% Hispanic) and parent dyads involved in a randomized-controlled trial of the FCU and followed longitudinally across 11 waves from ages 2 to 19. Results supported a developmental cascade from middle childhood physical aggression (parent reported) to self-reported legal system involvement at age 19. Early cumulative contextual risk predicted arrests (*b* = 0.35, *p* = .001), while a risk tolerance polygenic score predicted self-reported adolescent antisocial behavior (*b* = 0.51, *p* = .005). Further, the FCU attenuated the genetic effects on antisocial behavior in adolescence. Findings highlight the value of early prevention for reducing the impact of genetic and contextual risk on rates of justice system involvement by young adulthood.

According to the 2020 Juvenile Residential Facility Census, approximately 25,000 youth are currently incarcerated or placed in residential facilities within the U.*S*. juvenile justice system (Puzzanchera, 2022). While this number has been steadily decreasing from over 100,000 youth in 2000 to approximately 25,000 in 2020, costs associated with youth incarceration are still increasing. In 2020, the Justice Policy Institute reported that the average state costs associated with youth incarceration had risen 44% since 2014 to $588 per individual per day, equating to over $200,000 per year per youth (Sticker Shock, 2020: The Cost of Youth Incarceration, 2020).

Juvenile crime also results in substantial public health costs, including the physical, financial, and psychological costs that are incurred upon the victims. In fact, together tangible (e.g., damaged or stolen property, medical expenses, and loss of productivity) and intangible (e.g., increased fear, pain, and suffering leading to reduced quality of life) costs brought upon the victim by juvenile crime have been shown to be at least three times greater than those incurred by the criminal justice system (DeLisi, 2016; Welsh et al., 2008). Incarcerated youth and their families also face substantial personal costs, as justice system involvement in youth has been associated with poorer health and social outcomes (Young & Turanovic, 2022), including lower educational attainment due to disruption to their education (Liu et al., 2021) and less social support due to physical separation from their families. Furthermore, the burden of incarceration falls disproportionately onto youth of color. While youth of color make up only 34% of the U.S. population, they consist of 62% of the youth charged in the juvenile justice system (Dragomir & Tadros, 2020). Black African American youth are five times more likely to be incarcerated than White European youth, and youth of color are more likely to have any involvement with the criminal justice system and involvement that continues over time (Dragomir & Tadros, 2020).

Based on the clear individual, social, and economic costs of aggression and violence to society, research in the latter part of the 20^th^ century began to prospectively study both environmentally and biologically oriented risk factors to youth antisocial behavior. This work provided divergent theoretical frameworks (e.g., Moffitt, 1993; Patterson, 1982; Shaw & Bell, 1993) and the empirical identification of early risk factors for aggressive and dysregulated behavior (Moffitt et al., 1996; Nagin & Tremblay, 2001; Shaw et al., 2003, 2012) that underlie involvement with the criminal justice system, such as arrests and incarceration (Liu et al., 2013; Sitnick et al., 2017). Despite several advances in identifying mechanisms and risk factors underlying legal system involvement, additional research could help identify potential targets for early prevention programs and ultimately reduce the number of incarcerated youth. Such advances in our understanding could, in turn, help reduce the physical, social, and economic costs related to youth antisocial behavior and their incarceration. Thus, the current study sought to elucidate a developmental cascade from physical aggression and dysregulation in middle childhood to antisocial behavior in adolescence to involvement with the criminal justice system by early adulthood. Adopting a developmental psychopathology framework that considers the influence of both contextual and biological factors (Moffitt, 1993; Shaw & Bell, 1993), we examined how early childhood contextual and genetic risk may impact the developmental cascade (see Figure S1 in Supplementary Material), as well as whether an early intervention program could reduce the impact of risk on antisocial behavior and legal system involvement.

## Proposed developmental cascade

### Childhood aggression

High levels of aggressive behavior – defined as intent to harm or hurt another person (Anderson & Bushman, 2002) – across childhood and adolescence have consistently predicted greater risk for engagement in antisocial behavior and/or involvement with the criminal justice system (Huesmann et al., 2002; Moffitt, 1990). However, aggressive behavior is displayed differently across development, from childhood into adolescence and early adulthood (Bongers et al., 2004; Tremblay et al., 2018; Vitaro et al., 2006).

Physical aggression, often defined as the threat or infliction of physical damage, such as through (threatening) fighting, pushing, hitting, or kicking (Crick et al., 2006; Ersan, 2020), is normatively high in early childhood (ages 2–3), declines approaching age 5, and levels off across development, except for a select subset of children who continue to display high levels of aggressive behavior, often manifested as disruptive behavior and/or conduct problems. This subset of children is more likely to face negative societal consequences as a result of their highly aggressive behavior (Bongers et al., 2004; Tremblay et al., 2018), and this is associated with more serious forms of antisocial behavior in adolescence (Bongers et al., 2004; Shaw & Gross, 2008).

### Childhood dysregulation

Behavioral and emotional dysregulation – defined as difficulty managing if, when, and how one experiences emotions or expresses them behaviorally in the face of distress (Eisenberg et al., 2010; Roberton et al., 2012) – represents another potential risk factor for the development of antisocial behavior and criminal justice system involvement. The development of self-regulation typically begins at birth and continues throughout early childhood (Montroy et al., 2016; Rothbart et al., 2006). In fact, most children appear to experience rapid development of behavioral self-regulation before preschool and into kindergarten, except for some children who display lower behavioral self-regulation until around age 5 (Montroy et al., 2016). Liu et al. (2026) also found that increasing behavioral self-regulation, defined as aggression and attention problems from age 5 through adolescence (age 15), protects against the development of antisocial behavior compared to children who experienced higher levels of self-regulation at age 5. Overall, research has shown that behavioral dysregulation in early childhood is a prominent risk factor for the development of physical aggression in middle childhood and adolescence (Liu et al., 2013) and higher levels of antisocial behavior in adolescence (Liu et al., 2026).

### Adolescent antisocial behavior

Adolescents in general are more likely to engage in violent behavior, including the use of weapons, than younger children, resulting in a higher likelihood of injury or death to victims (Liu et al., 2013; Loeber & Hay, 1997). Adolescence also is marked by property violations and increases in status violations, such as running away, truancy, and risk taking (Bongers et al., 2004). This increase in antisocial behavior among adolescents may be partially attributed to the heritable trait of risk tolerance, or the degree that an individual engages with risk and uncertainty (Mann et al., 2017; Zuckerman, 1994). Adolescents may experience an increase in risk tolerance due to perceived benefits from peers, such as popularity, a sense of control or dominance, and even possible monetary gain (Bjorklund & Hawley, 2014; Liu et al., 2013). While risk tolerance may be beneficial in moderation, high risk tolerance has been associated with increased risky behaviors, sensation seeking, and impulsivity, which are heritable traits hypothesized to underlie antisocial behavior (Burt & Simons, 2013; Mann et al., 2017). Research findings confirm that peer pressure and group activities further compound adolescents’ risk of engaging in antisocial behavior, such as truancy, stealing, or gang violence (Bongers et al., 2004). In this way, a genetic propensity for high risk tolerance may reflect another influence on the development of antisocial behavior that may result in legal consequences (Karlsson Linnér et al., 2019; Wang et al., 2022), such as arrests and incarceration.

## Genetic and contextual influences on the proposed developmental cascade

### The early childhood context

Under the biopsychosocial model, aggressive behavior is influenced by interactions between biological factors, such as genetics, and environmental or contextual factors, such as family and peer influences (Raine, 2002; Salvatore et al., 2015; Tremblay et al., 2018). Contextual factors, such as harsh and unpredictable childhood contexts, have been associated with the development of aggression and other serious antisocial activities among youth (Chang et al., 2019; Loeber & Stouthamer-Loeber, 1986). Further, cumulative risk indices, such as Adverse Childhood Events (Felitti et al., 1998), examine the summative effects of exposure to multiple contextual risks beginning in early childhood. These adverse experiences, such as exposure to poverty, parent substance use, or having a parent incarcerated have been linked to higher levels of child disruptive behavior, antisocial behavior, and juvenile offending than exposure to a single risk factor (Evans et al., 2013; Jackson et al., 2023; Oei et al., 2023; Shaw et al., 1994; Shaw & Emery, 1988). This cumulative impact of exposure to multiple contextual risk factors may explain why sociodemographic factors, such as poverty, urbanicity, and neighborhood violence, have been associated with multiple types of adverse child and adolescent outcomes, including youth antisocial behavior (Evans et al., 2013; Loeber & Hay, 1997; Trentacosta et al., 2008).

While exposure to contextual risk, such as adversity, does change across development, early childhood serves as a sensitive period for development due to heightened neuroplasticity, including rapid neurodevelopment during the first two years of life (Gee, 2021; Nelson & Gabard-Durnam, 2020; Zhu et al., 2022). This sensitive period suggests that examining early childhood contextual risk as an indicator of early developmental exposure is important for informing early childhood prevention. Therefore, the current study examined whether early childhood contextual risk at age 2 (i.e., poverty status, neighborhood dangerousness, household overcrowding, parental education, parental incarceration, parental substance use, and teen and/or single parenthood) was associated with the developmental cascade from aggression and dysregulation in middle childhood to justice system involvement by early adulthood.

### Genetic influences

Importantly, most contextual risk factors, such as those examined in the current study, do reflect a degree of heritability, underscoring the importance of studying them together with genetic influences (Kendler & Baker, 2007). Further, aggression and antisocial behavior have been shown to be moderately heritable. Twin studies suggest that additive genetics account for at least 50% of individual variability in twin studies (Odintsova et al., 2019; Veroude et al., 2016) and at least 30% of variability for antisocial behavior (Baker et al., 2006; Gard et al., 2019). A polygenic score (PGS) is an aggregate of many small effects of genetic variants across the genome. Wang et al. (2022), also using the current sample, found that a cumulative risk index for adolescent antisocial behavior was only predictive for those who scored at high levels on a risk tolerance PGS. Outside of antisocial behavior, the PGS for risk tolerance has also been associated with Alcohol Use Disorder across two large samples (Barr et al., 2020). The current study examined the effects of a risk tolerance PGS on the proposed developmental cascade. Lastly, as other parenting-based programs initiated before child age 5 have been found to prevent the development of early-starting conduct problems during middle childhood (Sanders et al., 2000; Webster-Stratton & Hammond, 1997) and in some cases, more serious forms of antisocial behavior in adolescence (Olds, 2002), we were also interested in examining whether aggression developmental cascades might be attenuated by families’ engagement in an early childhood parenting intervention, the Family Check-Up (FCU).

## The Family Check-Up early prevention program

Intervention and prevention programs, such as parenting programs, have been created and implemented to target and prevent the development of conduct problems. In addition to programs like Parent-Child Interaction Therapy, Triple P, and the Incredible Years programs, an example of one of these interventions is the early childhood version of the FCU, a brief family-based program that incorporates motivational interviewing and evidence-based family management practices to increase positive parenting in families of children at high-risk of developing conduct problems (Dishion et al., 2008). In two randomized control trials (RCTs), the early childhood version of the FCU has demonstrated reductions in conduct problems through the preschool period (Brennan et al., 2012; Connell et al., 2008; Dishion et al., 2008; Shaw et al., 2006) and, in the present cohort, through middle childhood (Dishion et al., 2014; Shaw et al., 2016) and adolescence (Hentges et al., 2020; Shaw et al., 2019).

Studies have found that the FCU increases child inhibitory control (Chang et al., 2014) and reduces oppositional defiant behavior through age 7.5 at home and at school (Dishion et al., 2014) and results in less growth in antisocial behaviors from ages 2 to 14 relative to those in the control group (Shaw et al., 2019). Findings also suggest an indirect effect of the FCU on externalizing symptoms in adolescence through increased inhibitory control in middle childhood (Hentges et al., 2020). Recently, Tein et al. (2023) reported that the FCU reduced growth in a “*p*” factor of general psychopathology during early and middle childhood, which in turn resulted in reduced psychopathology and alcohol and drug use in adolescence. Further, Connell et al. (2012), in an independent sample of age 10–13 youth, found the FCU reduced the likelihood of arrest among those who exhibited increasing antisocial behavior in adolescence. Based on these direct effects of the FCU on arrest trajectories and indirect effects through inhibitory control, the current study tested the hypothesis that the FCU would attenuate the potential effects of cumulative contextual risk and genetic risk on antisocial behavior and arrests.

## Risk moderation by the Family Check-Up

### Cumulative contextual risk moderation

Other work with the current sample also suggests that the FCU moderates the effects of contextual factors on early childhood conduct problems, such as parent education level, single parenthood (Gardner et al., 2009), and neighborhood disadvantage (Shaw et al., 2016). FCU effects have been shown to be stronger in two-parent households and families with lower parental education. Importantly though, the FCU did not moderate the effects of other contextual risks, such as maternal depression, teen parent status, parent substance use, parenting daily hassles, and partner satisfaction (Gardner et al., 2009). The FCU also has been shown to directly reduce childhood aggression among children living in moderately disadvantaged neighborhoods and was indirectly effective for families living in neighborhoods marked by high deprivation through an increase in the quality of parent–child interactions (Shaw et al., 2016). Further, person-centered approaches suggest that the FCU is most effective at reducing child problem behaviors among families who were identified as lower income and at increased risk for child neglect, legal problems, and parental depression or mental health issues (Pelham et al., 2017). Therefore, the current study seeks to understand whether the FCU impacts the possible effects of age 2 cumulative contextual risk factors on the proposed developmental cascade.

### Polygenic moderation

Using the current sample and other trials of the FCU, a few studies have examined interactions between genetic risk and intervention group status (GxI), adding to a small but innovative literature (Shaw et al., 2006, 2016; Shelleby et al., 2018; Stormshak et al., 2019). In the current sample, Lemery-Chalfant et al. (2018) reported that children with high PGS for environmental sensitivity who were randomly assigned to the FCU condition displayed fewer symptoms of internalizing than children with high PGS in the control condition. Similarly, in the current sample, youth with high PGS for aggression in the FCU group were more likely to display persistently low conduct problems. In contrast, those with similar PGS within the control group were more likely to display persistently high conduct problems across ages 2 through 14 (Shaw et al., 2019). In a different FCU RCT initiated in adolescence, individuals with higher alcohol dependence PGS in the control group were more likely to receive a diagnosis, whereas this association was not found for those with higher alcohol dependence PGS in the intervention group (Kuo et al., 2019). With this same adolescent sample, Elam et al. (2021) reported a significant GxI between higher PGS for aggression and increased likelihood of receiving a diagnosis of substance use disorder by the age of 27 through legal violations involving substance use, such that the simple slope for those who did not receive the FCU (i.e., the control group) was significant while the slope for those who were assigned to the FCU was not significant (Elam et al., 2021). Despite this work, no study has examined whether polygenic risk moderates the association between the FCU and antisocial behavior.

Therefore, the current study sought to examine the potential effects of both contextual risk and polygenic risk on the developmental cascade within the same model to elucidate whether both contextual risk and polygenic risk provide unique contributions to the developmental cascade, above and beyond the effects of the other. To reduce the number of statistical tests, we focused on polygenic and contextual risk effects on the mediators (e.g., adolescent antisocial behavior), as the risk tolerance PGS was explicitly geared towards adolescence. In addition, genetic effects have been shown to become more salient with age as adolescents begin to select their own environments (Abdellaoui et al., 2022), and legal involvement in young adulthood would theoretically be more strongly influenced by social and contextual factors, considering the racial disparities within the criminal justice system and society.

## The present study

The present study modeled relations between cumulative contextual risk and genetic risk tolerance through developmental cascading mediation, while examining whether cumulative contextual risk and polygenic risk tolerance serve as distal risk factors for involvement with the criminal justice system (see Supplementary Material Figure S1 for conceptual model). For the cascading effects, we hypothesized that (H1) higher physical aggression and dysregulation in middle childhood would predict higher levels of antisocial behavior in adolescence and higher rates of legal trouble by early adulthood. Further, regarding distal risk, we hypothesized that (H2) genetic and early contextual risk would have independent positive associations with childhood aggression and dysregulation, antisocial behavior in adolescence, and legal involvement by age 19. Finally, we also hypothesized that the potential effects of (H3a) age 2 cumulative contextual risk or (H3b) genetic risk on childhood aggression and dysregulation, antisocial behavior, and legal involvement would be attenuated for youth who were randomly assigned to the FCU intervention in early childhood.

## Methods

### Participants

This was a secondary data analysis study that included 731 caregiver-child dyads who were recruited for the Early Steps Multisite Study, an RCT of the FCU, an intervention designed to increase positive parenting and prevent child conduct problems (see Dishion et al., 2008). The racially/ethnically diverse, low-income families were recruited when the children were two years old from Women, Infant, and Children Nutritional Supplement (WIC) programs in urban (Pittsburgh, PA; *n* = 271, 37%), suburban (Eugene, OR; *n* = 271, 37%), and rural (Charlottesville, VA; *n* = 188, 26%) areas. Families were eligible to participate in the study if they scored >1 *SD* in at least two out of three domains: family problems (i.e., maternal depression, teen parent when first child was born, daily parenting challenges, parent substance use, or mental health diagnosis), sociodemographic risk (i.e., low parental education, low family income), or child behavior problems (i.e., conduct problems, high-conflict relationship). To increase parents’ motivation to engage in the FCU, for families not qualifying based on the presence of early child behavior problems, scores above the mean on at least one of the two Eyberg Behavior Inventory factors (Robinson et al., 1980; Webster-Stratton, 1985) were required for eligibility.

At the baseline assessment (age 2), the vast majority (97%) of primary caregivers were mothers, and 58% of children lived in two-parent households. Primary caregivers reported on children’s (49.5% female) race/ethnicity: 50.1% White/European American, 27.9% Black/African American, 13.0% Latino/Hispanic, 13.0% biracial, and 8.9% different groups (e.g., American Indian; Native Hawaiian). Almost a quarter (27%) of primary caregivers had less than a high school education, 41% had a high school diploma or general education diploma (GED), and 32% had 1–2 years of post-high school education. More than two-thirds of families reported an annual income of less than $20,000 at age 2 (see Dishion et al., 2008 for additional study and sample information).

At age 19, the participating youth (*N* = 536) reported on their own birth sex (53.1% female, 45.3% male) and race/ethnicity: 58.1% White/European American, 40.2% Black/African American, 11.7% Latino/Hispanic, 21.2% biracial, and 7.8% different groups (e.g., American Indian; Asian). Further, almost three-quarters (73.6%, *N* = 395) of the youth completed high school or a GED, while only 35.3% of the youth were currently in school either full-time (25%) or part-time (10.2%). Data collection at age 19 was during the COVID-19 pandemic shutdowns which may have affected demographic factors, including that 66.3% (*n* = 356) of youth were living with their parents.

### Procedures

All study waves were approved by the University’s Human Subjects Institutional Review Boards, and parents/guardians provided informed consent prior to the youth turning 18. Youth also provided assent beginning at age 14 and informed consent beginning at age 18. After enrollment and before the baseline assessment at age 2, families were randomly assigned to either the intervention (367 families) or the control condition (364 families), which consisted of WIC program services as usual (see CONSORT diagram; Figure 1). Families were followed up when children were 3, 4, 5, 7.5, 8.5, 9.5, 10.5, 14, 16, and 19 years old for assessments, and families in the intervention condition were offered the FCU nearly annually through child age 10.5. A representative sample of the participants (*n* = 515) also provided saliva samples at the age 14 home visit using Oragene kits for genotyping. There were no significant differences between the full sample and the genotyped sample with respect to ethnicity, gender, age, or study site. All participants were financially compensated following each assessment. The present study utilized observational and survey data collected from primary caregivers and youth during in-home assessments, except at age 19 when data were collected through online surveys and during a telephone interview.

### The Family Check-Up prevention program

The FCU is a brief, three-session intervention designed to encourage positive parenting with the intention of preventing child conduct problems (Dishion et al., 2008). The intervention was embedded in motivational interviewing (Miller & Rollnick, 2012) to promote change in caregiving practices among parents reporting high levels of early-starting conduct problems in their toddler-age children. The FCU uses motivational interviewing to generate dissonance between parents’ perception of child problem behavior and their aspirations for their child, thereby promoting change in caregiving. Evidence-based parenting management practices are then used to show parents alternative ways to respond to child misbehavior and proactively “catch their children being good,” capitalizing on parents’ existing strengths to promote child prosocial behavior. The FCU typically begins with an initial get-to-know-you meeting that orients parents to the FCU. The family coach then inquires about family values/culture and the parents’ aspirations for the child. This initial interview is followed by an ecological assessment of family and child behavior in which observational measures of parent-child relationship quality are videotaped for later coding by the family coach. In the third session, the family coach provides feedback to the parent(s), describing family strengths and challenges based on data from the initial interview and the assessment sessions, including positive video clips from the observational data recorded at the assessment. Parents then have the option of working on goals with or without the family coach during follow-up treatment sessions. To reduce bias in intervention families having contact with the FCU coach prior to the assessment, the assessment occurred prior to the initial interview in the current study.

Families received monetary compensation for their participation in the home visit assessments. Families offered the FCU received an additional $25 for completing the feedback session but no other financial compensation for completing the FCU or follow-up treatment sessions. Families in the intervention condition were annually offered follow-up FCUs at child ages 2–5 and 7.5–10.5, but participation varied by child age as families randomly assigned to the FCU were able to decide whether to receive the intervention at each age. For those in the FCU intervention condition, the percentage of families who completed at least a feedback session ranged from 56.7% to 77.7% from child ages 2 through 5 (with 90% of intervention families having at least one completed FCU with a feedback session), and 46.0% to 55.3% from child ages 7.5 to 10.5. Although the default was to maintain continuity in having the same FCU coach work with the same families across the child’s development, because of FCU coach attrition, in some cases, families who completed multiple feedback sessions across years may have worked with different FCU coaches; however, fidelity to the Family Check-Up in the current sample was high, and interrater reliability for fidelity coding was acceptable (Smith et al., 2013). In the current study, the FCU (0 = *control*, 1= *FCU intervention*) was controlled for in H1 and H2 and considered a developmental moderator for H3.

### Measures

#### Early contextual risk

Cumulative contextual risk was operationalized as a cumulative risk index (Trentacosta et al., 2008) that represented a sum score from eight binary indicators of the child’s exposure to specific contextual risk factors at the age of 2: teen parent status, parent education level, being a single parent, household overcrowding, household member legal conviction, parent substance use, neighborhood dangerousness, and poverty status. This early childhood cumulative risk index has been shown to be indirectly associated with child externalizing and internalizing problems at age 4 through observed parental nurturance and involvement assessed at child age 3 (Trentacosta et al., 2008).

#### Polygenic risk tolerance

A PGS for risk tolerance was computed utilizing thresholding. See Wang et al. (2022) for an explanation of how the risk tolerance PGS was formed based on summary statistics from a meta-GWAS of general risk tolerance in the UK Biobank and a replication GWAS across ten smaller cohorts from seven studies (Karlsson Linnér et al., 2019). After cleaning and clumping to control for multicollinearity, 218,779 Single Nucleotide Polymorphisms (SNPs) overlapped between our sample and the meta-GWAS. To reduce the number of models, only the effect size *p*-value threshold of *p* < .05 (*N* = 33,673 SNPs) was tested.

#### Genetic ancestry control variables.

To account for systematic genetic variation due to ancestry, principal components analysis was performed on non-imputed genetic data using PLINK 1.9.21 (www.cog-genomics.org/plink/1.9/), and the first 20 principal components (PCs) were extracted (Price et al., 2006). The effects of the 20 PCs on the risk tolerance PGS were regressed out prior to analyses.

#### Behavioral/emotional dysregulation

Behavioral/emotional dysregulation in middle childhood was assessed observationally at ages 7.5 (*n* = 526), 8.5 (*n* = 521), 9.5 (*n* = 546), and 10.5 (*n* = 504) on the Behavioral/Emotional Dysregulation subscale of the Moffit Lack of Control measure (Caspi et al., 1995). Examiners recorded their impressions of children’s behavioral/emotional dysregulation across five items (e.g., emotional liability, frustration tolerance, hostility, roughness, resistance) at the end of home-based assessments at ages 7.5, 8.5, 9.5, and 10.5 on three-point Likert-type scales (“Not at all,” “Somewhat,” “Definitely”.) The behavioral/emotional dysregulation mean composite, shown to be longitudinally positively related to externalizing problems and fewer competencies (Caspi et al., 1995), demonstrated strong internal consistency across ages 7.5 to 10.5 in the current sample, with Cronbach’s αs ranging from 0.79 to 0.88.

#### Childhood physical aggression

Parent-report of middle childhood aggression was measured through the 18-item aggressive behavior subscale of the Child Behavior Checklist for Ages 6–18 (CBCL) at ages 7.5, 8.5, 9.5, and 10.5 (Achenbach & Rescorla, 2001). Parents rated their child using a three-point Likert-type scale ranging from “Not true” to “Very true or often true” with higher scores indicating greater aggression. Example items include “Argues a lot,” “Disobedient,” “Cruelty, bullying, or meanness to others,” “Physically attacks people,” “Screams a lot,” and “Explosive or unpredictable behavior.” The CBCL aggression subscale had strong internal reliability for the current sample with Cronbach’s αs ranging from 0.91 to 0.92 at ages 7.5, 8.5, 9.5, and 10.5.

Parent-report of age 2 aggression was also measured through the 19-item aggressive behavior subscale on the CBCL for Ages 1.5–5 (Achenbach & Rescorla, 2000; Achenbach, 1992). This measure utilized the same Likert-type scale but differed in the content of the questions to match developmental expectations of early childhood. Example items include “Demands must be met immediately,” “Punishment doesn’t change their behavior,” “Uncooperative,” and “Temper tantrums or hot temper.” The internal reliability of the CBCL aggression subscale at age 2 was a Cronbach’s *α* of 0.85.

#### Adolescent antisocial behavior

Youth’s antisocial behavior was assessed using youth self-report at ages 14 and 16 and subsequently modeled as a latent construct in a confirmatory factor analysis. Indicators of the latent factor were drawn from a modified version of the Self-Report of Delinquency (Elliot et al., 1985). It consists of 27 items rated on a three-point Likert scale (“Never,” “Once or twice,” “Often”) to assess how frequently youth have engaged in aggressive and antisocial activities in the past year. Sample items based on the past year include questions such as “Have you snatched someone’s purse or wallet or picked someone’s pocket?,” “Have you threatened anyone with a weapon (like a bat, brick, broken bottle, knife, or gun)?,” and “Have you hit other students or got into a physical fight with them?.” Items assessing substance use were omitted, as substance use was assessed elsewhere. The Self-Report of Delinquency is reliable and valid for at-risk populations (Huizinga & Elliott, 1986; Pechorro et al., 2019), and had good internal consistency for the current sample (*α* = .85 at age 14 and *α* = .86 at age 16).

#### Adolescent callous-unemotional traits

Because callous-unemotional traits are conceptually related to antisocial behavior and were included in the confirmatory factor analysis used to derive the latent antisocial behavior construct, callous-unemotional traits were also assessed at ages 14 and 16 using a shortened, 12-item version of the Inventory of Callous-Unemotional Traits (ICU; Frick, 2004). The ICU consists of three subscales, each measuring a dimension of callous-unemotional traits: callousness, uncaring, and unemotionality. Youth rated each item on a four-point Likert scale ranging from “not at all true” to “definitely true.” The ICU has been shown to be reliable and valid (Essau et al., 2006), and the internal consistency of the ICU for the current sample was adequate (*α* = .77 at age 14 and *α* = .79 at age 16).

#### Emerging adult trouble with the law

To assess overall involvement with the justice system, youth reported the extent to which they had been in trouble with the law at the age 19 assessment. Self-reports of arrests have been shown to be reliable and valid, providing substantial consistency with both parent-report and public criminal court records (Gomes, 2025; Holloway et al., 2024; Maxfield et al., 2000; Pollock et al., 2015). Youth were asked the yes/no question, “Have you EVER had trouble with the law?.” If they had been in trouble with the law, target youth also reported the time of the crime or incarceration (e.g., whether the offenses were committed as a juvenile, an adult, or both), what offenses they were charged with, whether they served time for the charges, and, if so, where/how long they served time. A subsample of youth (*n* = 64) reported the charges they faced, most commonly arrest-related offenses (*n* = 29; e.g., drug possession, driving under the influence, disorderly conduct), followed by driving or vehicle violations (*n* = 12). Less frequent charges included fraud (*n* = 1), truancy (*n* = 3), and curfew violations (*n* = 1). Some youth reported no applicable charges (*n* = 2), refused or did not know their charges (*n* = 8), or reported other reasons for legal trouble (*n* = 8). Two dichotomous (0 = no, 1 = yes) variables for trouble with the law and incarceration were created. For ease of interpretation, youth were conceptualized as having been arrested (i.e., apprehended by law enforcement) if they indicated “yes” to having been in trouble with the law and as having been incarcerated if they reported serving time for legal charges. Youth who spent time on house arrest were considered to have been in trouble with the law but not incarcerated.

#### Covariates

Sociodemographic covariates included birth sex (0 = *female*, 1= *male*), race (indexed with multiple dummy codes), youth age at the age 19 assessment, and *z*-scored household monthly income at age 16. Household monthly income at age 16 was chosen, as this represents the youth’s household income at a salient time for the potential developmental cascading effects of antisocial behavior on legal system involvement (household monthly income at age 2 was not used due to concerns about multicollinearity, as parent poverty status at age 2 (i.e., having an income below the poverty line at the time) was included in the cumulative risk index and was highly correlated with household monthly income at age 2 (*r* = −.708, *p* < .001). Household monthly income at age 19 was not used, as there was variability in how youth reported it, and data collection occurred during pandemic shutdowns). Further, parent-report of aggression on the CBCL at age 2 was included as a covariate to control for baseline levels of aggression. Random assignment into the intervention condition (0 = *control*, 1= *FCU intervention*) was also treated as a covariate in H1 and H2.

### Data analysis

Analyses were completed using Mplus Version 8.8 (Muthén & Muthén, 1998–2017). To address missing data, full information likelihood was used (Enders, 2022) for all analyses. Structural equation modeling (SEM) was conducted to account for the covariance between the predictors and analyze the variables in the models simultaneously. All study variables were assessed for normality. Skewed variables were handled through maximum likelihood with robust standard errors (MLR). The PGS and monthly household income at age 16 were *z*-scored.

We formed middle childhood physical aggression and dysregulation composite scores if the scores across the assessments were stable. We first tested stability of the longitudinal predictors (e.g., aggression and dysregulation) across the four time points (i.e., ages 7.5, 8.5, 9.5, and 10.5) using Stable Trait, Autoregressive Trait, and State (STARTS) models (see Supplementary Material S1; Donnellan et al., 2012; Kenny & Zautra, 2001). If the stable trait of the predictors (e.g., manifest variables for childhood aggression and dysregulation) accounted for a majority of the variance in the model, then composite variables for each construct in the cascade model were generated for use in the SEM path analysis. Composite variables, rather than latent constructs generated by the STARTS models, were used in path analysis of the developmental cascade to preserve parsimony for ease of interpretation and replication. The common factors with the four assessments of the same variables as the indicators were examined through confirmatory factor analysis (CFA). Further, a latent factor representing antisocial behavior at ages 14 and 16 was examined through a CFA. Consistent with prior research conceptualizing antisocial behavior and callous-unemotional traits as related but distinct dimensions of aggression or disruptive behavior, a correlated two-factor CFA was specified in which the two antisocial behavior indicators loaded on one latent factor and the two callous-unemotional indicators loaded on a second latent factor. This measurement model was used to evaluate the discriminant validity of the antisocial behavior construct relative to callous-unemotional traits. Model fit for the CFA was determined based on chi-square difference tests, RMSEA, loglikelihood and information criteria, and SRMR. Factor scores from the antisocial behavior latent factor were saved and used in the path analyses.

Following factor score formation and extraction, path analyses were conducted. Direct and indirect paths were tested simultaneously according to best practices for testing cascade models (Masten & Cicchetti, 2010; Scheier & Shigeto, 2022). As the age 19 outcome variables – arrests and incarceration – were dichotomous, SEM path analyses for H1 and H2 were specified with the MLR estimation method, as MLR calls for logistic regression with a logit linking function. To test H3, multigroup path analyses were run in Mplus according to Muthén and Muthén’s procedures (1998–2017). The weighted least squares mean- and variance-adjusted (WLSMV) estimator and a probit linking function was used for testing H3. Multigroup path analyses across FCU assignment (control vs. intervention) were run in Mplus using multigroup longitudinal SEM, controlling for age, sex, racial/ethnic identity, income, and age 2 aggression. Within the multigroup path analyses, the statistical differences in the parameter estimates across the two groups (control vs. intervention) for the hypothesized mediation paths were tested by examining whether the parameters in the control vs. intervention condition were significantly different from each other (Muthén & Muthén, 1998–2017).

## Results

Frequencies for the self-report of criminal justice involvement are reported in Table 1. Overall, most youth reported having never been arrested (78.2%, Table 1a) or incarcerated (83.0%, Table 1b); however, a small percentage (6.3%, *n* = 34) had been incarcerated multiple times. Race (White vs Non-White and Black vs Non-Black) was not associated with age 2 baseline aggression (see Supplementary Material Table S1). Importantly, 90.9% of the sample reached criteria for at least one cumulative contextual risk factor at age 2, with the majority experiencing two or three (53.1%).

Descriptive statistics and bivariate correlations for study variables included in the SEM models are reported in Supplementary Material Table S1. Cumulative contextual risk at age 2 and adolescent antisocial behavior were positively correlated with the risk tolerance PGS. Prior to controlling for the ancestry PCs, the risk tolerance PGS was significantly associated with ancestry PC1, ancestry PC2, and the dummy-coded race variables (White vs. non-White and Black vs. non-Black), but these associations were not significant once the 20 PCs were regressed out of the PGS. Cumulative contextual risk at age 2 was modestly but positively correlated with aggression at age 2 and self-report of criminal justice system involvement at age 19 but not associated with middle childhood aggression or dysregulation or adolescent antisocial behavior. Middle childhood aggression had significant positive correlations with middle childhood dysregulation and adolescent antisocial behavior. Middle childhood aggression and adolescent antisocial behavior were also significantly correlated with age 2 aggression. Further, middle childhood aggression and latent adolescent antisocial behavior, but not middle childhood dysregulation, were positively correlated with criminal justice system involvement, including arrests and incarcerations. There was also a weak, positive correlation between being arrested, but not incarcerated, and age 2 aggression.

### Composite variables

Supplementary Material Figures S2–S3 show the results of the STARTS models, testing stability of the longitudinal predictors (e.g., physical aggression and behavioral/emotional dysregulation) across the four time points (i.e., ages 7.5 to 10.5). The results showed that the stable trait accounted for a majority of the variance in the model, indicating that aggression and dysregulation were relatively stable across middle childhood. With confidence in the stability of aggression and dysregulation across middle childhood development, a latent composite for each was formed using CFA. The manifest variables of aggression and dysregulation at each age in middle childhood were significantly correlated (see Table S2 in Supplementary Material). The factor loadings for aggression were significant (λs = 0.812 – 0.891, *p* < .001), while the factor loadings for dysregulation displayed more variation (λ_7.5_ = 0.595, *p* < .01; λ_8.5_ = 0.801, *p* < .05; λ_9.5_ = 0.573, *p* < .05; λ_10.5_ = 0.582, *p* = ns). The CFAs for aggression and dysregulation fit reasonably well (Aggression: *AIC* = 13,779.70; *BIC* = 13,842.33; *RMSEA* = 0.0 [0.0, 0.0], *CFI* = .998, *TLI* = 1.00, *SRMR* = .004; Dysregulation: *AIC* = −596.33; *BIC* = −534.54; *RMSEA* = 0.0 [0.0, 0.0], *CFI* = .897, *TLI* = 1.00, *SRMR* = .022). The composites for aggression and dysregulation were highly correlated with the stable trait constructs from the STARTS models (*r* = .990 and .965 respectively, *p* < .001).

For antisocial behavior, a correlated two-factor CFA with callous-unemotional traits demonstrated acceptable fit (*AIC* = 8,319.48; *BIC* = 8,381.01; *RMSEA* = 0.0 [0.0, 0.0], *CFI* = 1.0, *TLI* = 1.0, *SRMR* = 0.0). The manifest variables for adolescent-reported antisocial behavior and callous-unemotional traits at ages 14 and 16 were significantly correlated (see Supplementary Material Table S3). The adolescent antisocial behavior and callous-unemotional factors were moderately correlated (*r* = .54, *p* < .001), indicating that the constructs were related but distinct. The antisocial behavior factor loadings were significant (λ_14_ = 0.613 and λ_16_ = 0.893, *p* < .001). Because the study hypotheses focused on antisocial behavior, factor scores from the antisocial behavior latent factor were saved and used in subsequent path analyses.

### Developmental cascade effects

Figure 2 shows the cascade model from middle childhood aggression and dysregulation to adolescent antisocial behavior to young adulthood arrests. The unstandardized regression coefficients for the significant paths are indicated, excluding those for the direct paths and covariates for simplicity. Partially supporting H1, aggression in middle childhood, but not dysregulation, positively predicted antisocial behavior in adolescence, which significantly predicted arrests in young adulthood.

### Genetic and contextual risk

To test H2, the associations of polygenic and cumulative contextual risk were added to the cascade path model predicting arrests. The results are reported in Figure 3. The developmental cascade effects remained the same, with middle childhood aggression predicting adolescent antisocial behavior, which in turn predicted early adult arrests. Cumulative contextual risk was a significant direct positive predictor of arrests. The risk tolerance PGS was a significant negative predictor of middle childhood aggression and, simultaneously, a significant positive predictor of adolescent antisocial behavior. Overall, H2 was partially supported.

### Risk moderation of the intervention

Multigroup comparisons of the full cascade model across the intervention and control groups were conducted and reported in Figure 4. Contrary to H3a, the effects of cumulative contextual risk on arrests did not significantly differ across the two groups, although the path was significant in the FCU group.

Supporting H3b, there was a significant difference between the effect of the risk tolerance PGS on antisocial behavior between the intervention and control groups. In the control group, the risk tolerance PGS significantly positively predicted antisocial behavior. However, among those assigned to the FCU, the risk tolerance PGS did not significantly predict adolescent antisocial behavior (see Figure 4; difference in unstandardized parameter estimates across groups: *b* = 0.72, *SE* = 0.36, *p* = .04). This finding suggests that receiving the FCU attenuated the genetic impact on antisocial behavior in adolescence.^1^

## Discussion

This study examined the longitudinal cascading effects from middle childhood physical aggression and dysregulation to adolescent antisocial behavior to involvement with the law by young adulthood. In addition, the present study sought to elucidate the effects of cumulative contextual and polygenic risk on this developmental cascade and whether the effects of cumulative contextual and polygenic risk would differ according to families being randomly assigned to the FCU prevention program in early childhood.

### Developmental cascade of trouble with the law

As expected, and consistent with prior research (Kyne, 2020; Shaw & Gross, 2008), middle childhood aggression, but not behavioral and emotional dysregulation, significantly predicted antisocial behavior in adolescence, which in turn predicted arrest by age 19, even when controlling for sex at birth, race, monthly income, and age 2 aggression (H1). This finding strengthens prior literature by examining the longitudinal pathway from aggression in middle childhood to justice system involvement by age 19, while accounting for baseline aggression at age 2, thereby spanning 17 years of development. Further, antisocial behavior in adolescence mediated the association between middle childhood aggression and young adult arrests, adding new specificity to the literature. Observed childhood behavioral and emotional dysregulation did not significantly predict adolescent antisocial behavior, suggesting that dysregulation may operate through mechanisms not tested by the current developmental cascade.

### Cumulative contextual risk on justice system involvement

The current study also found some support for H2. Cumulative contextual risk at age 2 significantly predicted young adult criminal justice system involvement, even after accounting for the developmental cascade, polygenic risk, intervention group, race, sex at birth, income, age, and baseline aggression at age 2. This finding highlights cumulative contextual risk as a salient predictor of long-term outcomes and bolsters previous literature suggesting effects of cumulative contextual risk on longitudinal antisocial outcomes are often mediated by other factors, including dimensions of parenting (Gach et al., 2018; Trentacosta et al., 2008), peer relationships, and neighborhood disadvantage (Burt, 2022).

Our study also found that the effects of cumulative contextual risk on arrests did not differ across random assignment to intervention or control group (H3a) but remained significant only in the FCU group. This contrasts with prior work showing FCU effects moderated by specific contextual risks, including parent education level, single parenthood, (Gardner et al., 2009) and neighborhood disadvantage (Shaw et al., 2016). For example, Gardner et al. (2009) found stronger FCU effects on early conduct problems among two-parent households and families with lower parental education; however, it should be noted that even in this study, there were seven other factors not found to moderate treatment response. Another study found direct effects of the FCU on middle childhood aggression for families in moderately disadvantaged neighborhoods, with indirect effects through early childhood positive parental engagement among families living in neighborhoods marked by extreme deprivation (Shaw et al., 2016). Therefore, while the current study finds that cumulative contextual factors at age 2 have a direct effect on arrests among those assigned to the FCU in early childhood, unmeasured or more proximal factors may also shape this association.

### Polygenic risk effects and the Family Check-Up

The effects of the risk tolerance PGS on later antisocial behavior was linked to intervention group status. As hypothesized, the risk tolerance PGS positively and significantly predicted adolescent antisocial behavior (H2; Figure 3) but only in the control group (H3b; Figure 4). Building on the work of previous studies finding genetic moderation of the effects of the early childhood FCU (Elam et al., 2022, 2025; Lemery-Chalfant et al., 2018; Shaw et al., 2019), evidence of genetic prediction only in the control group suggest that the FCU intervention attenuated genetic effects on adolescent antisocial behavior, even with the additional controls of the developmental cascade, race, sex at birth, income, and age. These findings are consistent with theories of environmental compensation (Shanahan & Hofer, 2005) and differential susceptibility (Belsky et al., 2022; Belsky, 2013). By increasing positive parenting practices in early childhood, the FCU may reduce the expression of genetic vulnerability associated with risk tolerance, particularly among youth with higher polygenic risk.

### Strengths, limitations, and future directions

Although participants were individually randomized, some degree of non-independence may have existed among families recruited within the same study site. Recruitment site was included as a covariate to account for differences across sites. However, future multisite trials with a larger number of sites should consider intraclass correlation coefficients across sites, and if high enough, using analytic approaches that account for clustering across sites. An additional limitation pertains to the PGS analyses. PGS, which are derived from GWAS and meta-GWAS summary statistics, are primarily based on European-origin populations, impacting the generalizability of the findings. Given that the current sample is racially/ethnically diverse, the PGS’s predictive validity in the current sample may be limited, even after accounting for genetic ancestry using 20 principal components. Future research should continue working to improve the generalizability of PGS scores to diverse samples through increasing the ancestral diversity of the GWAS discovery samples.

Further, while a strength of the current study is the high-risk community-based sample, findings from studies with greater variability in the degree of criminal justice system involvement would aid in elucidating the mechanisms underlying criminal justice system involvement. Similarly, while self-reports have been shown to be useful and valid (Gomes, 2025; Holloway et al., 2024; Maxfield et al., 2000; Pollock et al., 2015), future work would benefit from examining public records of arrests and incarcerations rather than rely on self-reports. The current study also examined contextual risk exclusively at age 2 due to evidence suggesting the importance of contextual risk in early childhood; however, examining the effect of contextual risk across development, including potential changes across time, would be an important extension of this work. Strengths of the current study include the racial/ethnic diversity of the sample and its longitudinal design, which allow for the examination of cascading effects across 17 years of development.

Future studies should seek to replicate these findings or add to them by examining criminal justice system involvement beyond age 19. Examining whether recidivism rates are influenced by behavior, contextual risk, genetic risk, or societal biases would be particularly salient. Given that the current study found that a positive parenting intervention significantly buffered polygenic risk, future research should also examine other potential protective factors in this developmental cascade and mechanisms that buffer the path from early childhood cumulative contextual risk to involvement with the law in early adulthood. Additional research is also being done to increase the scalability of the FCU and promote implementation, such as Stormshak and colleagues’ development of an online adaptation of the FCU (2019) and multiple applications in primary health care settings (Berkel et al., 2021; Shaw et al., 2024; Weaver Krug et al., 2024).

### Conclusion

Findings support a developmental cascade predicting criminal justice system involvement, which is influenced by polygenic risk tolerance. Further, cumulative contextual risk at age 2 was associated with justice system involvement by age 19. In contrast, the FCU, a positive parenting intervention, attenuated the effect of polygenic risk tolerance on the development of antisocial behavior. These findings provide additional evidence that psychosocial interventions can buffer polygenic risk, supporting the benefit and utility of early psychosocial prevention programs. Importantly, implementing programs that prevent youth arrests and incarceration would aid in reducing the state and federal fiscal costs within the criminal justice system (Sticker Shock, 2020: The Cost of Youth Incarceration, 2020), while also reducing the social and economic costs faced by families of incarcerated youth, as well as the victims of youth violence (Dragomir & Tadros, 2020). Overall, the findings of this study have important implications for both psychological prevention programs and society as a whole, in particular the criminal justice system and public policy.

## Supplementary Material

Supplementary Material

**Supplementary material.** The supplementary material for this article can be found at https://doi.org/10.1017/S0954579426101898.

## Figures and Tables

**Figure 1:**
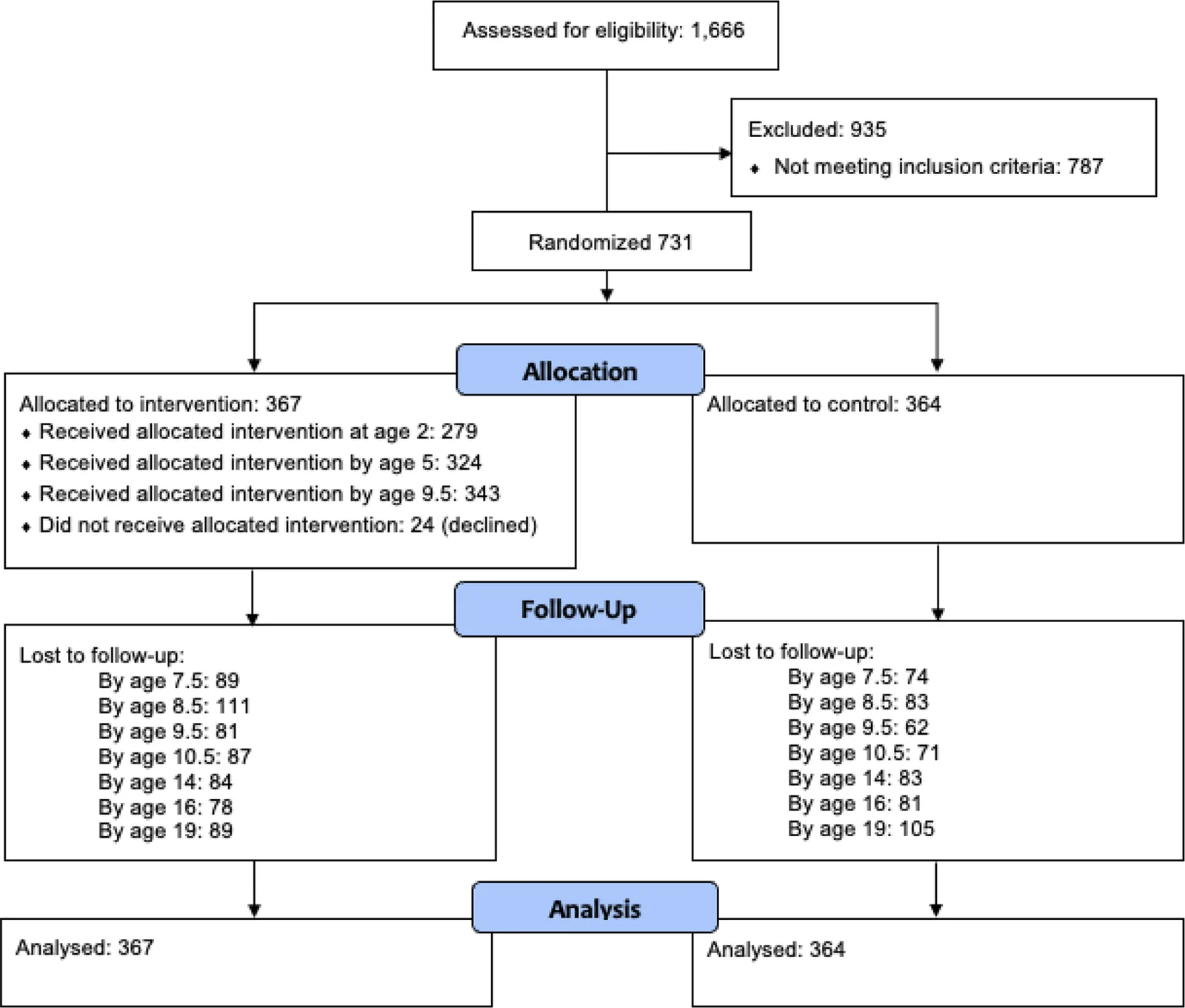
CONSORT 2010 Flow Diagram for the Early Steps Multisite Study *Note*. More information on engagement in the intervention have been reported by Smith and colleagues (2018). All individuals in the sample were included in the analysis using Full Information Maximum Likelihood (FIML) Estimation in Mplus.

**Figure 2:**
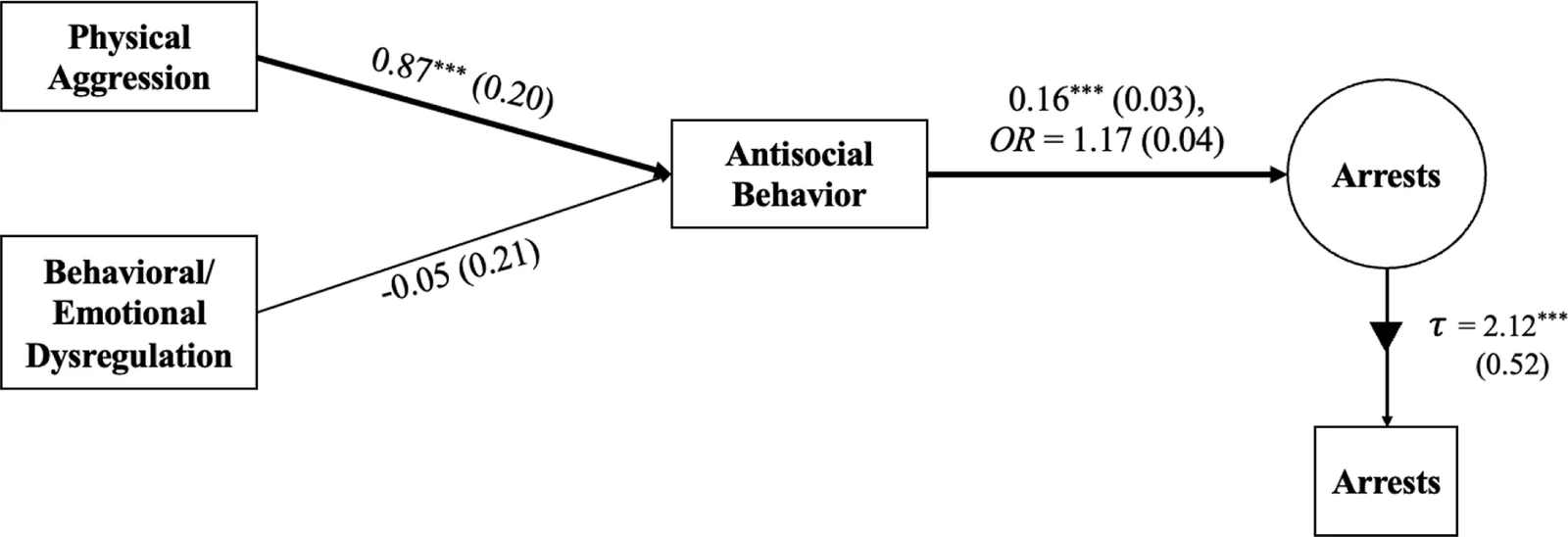
Developmental Cascade from Childhood Aggression and Dysregulation to Adolescent Antisocial Behavior to Young Adult Trouble with the Law *Note*. Direct paths and covariates, including intervention group, sex at birth, age, race, and monthly income, were included but not depicted for simplicity. * *p* ≤ .05. ** *p* ≤ .01. *** *p* ≤ .001.

**Figure 3:**
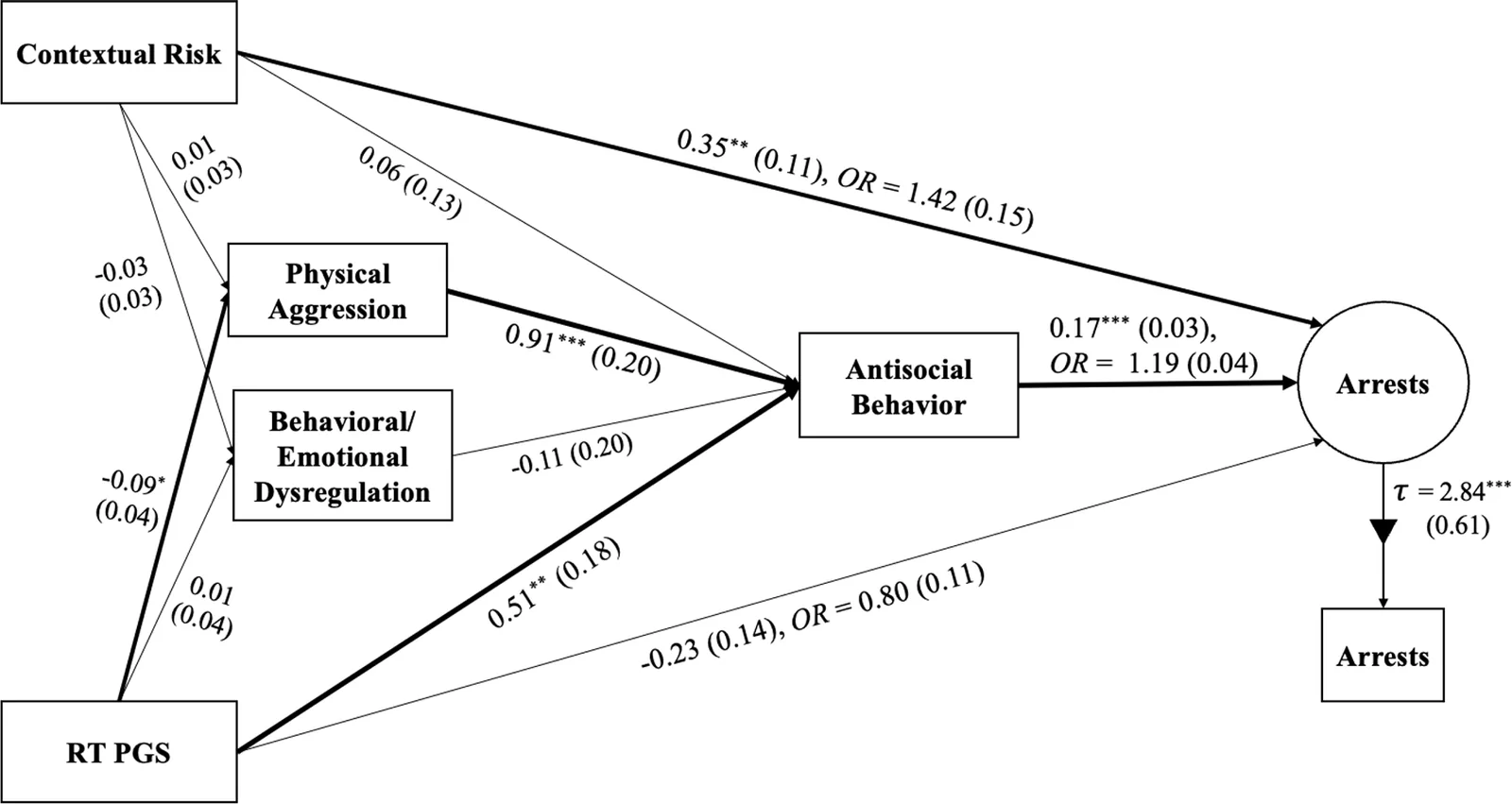
Polygenic Risk Tolerance and Early Childhood Cumulative Contextual Risk: Direct and Indirect Effects on Young Adult Trouble with the Law *Note*. RT PGS = polygenic risk score for risk tolerance. Direct paths and covariates, including intervention group, sex at birth, age, race, monthly income and baseline aggression at age 2, were included but not depicted for simplicity. * *p* ≤ .05. ** *p* ≤ .01. *** *p* ≤ .001.

**Figure 4:**
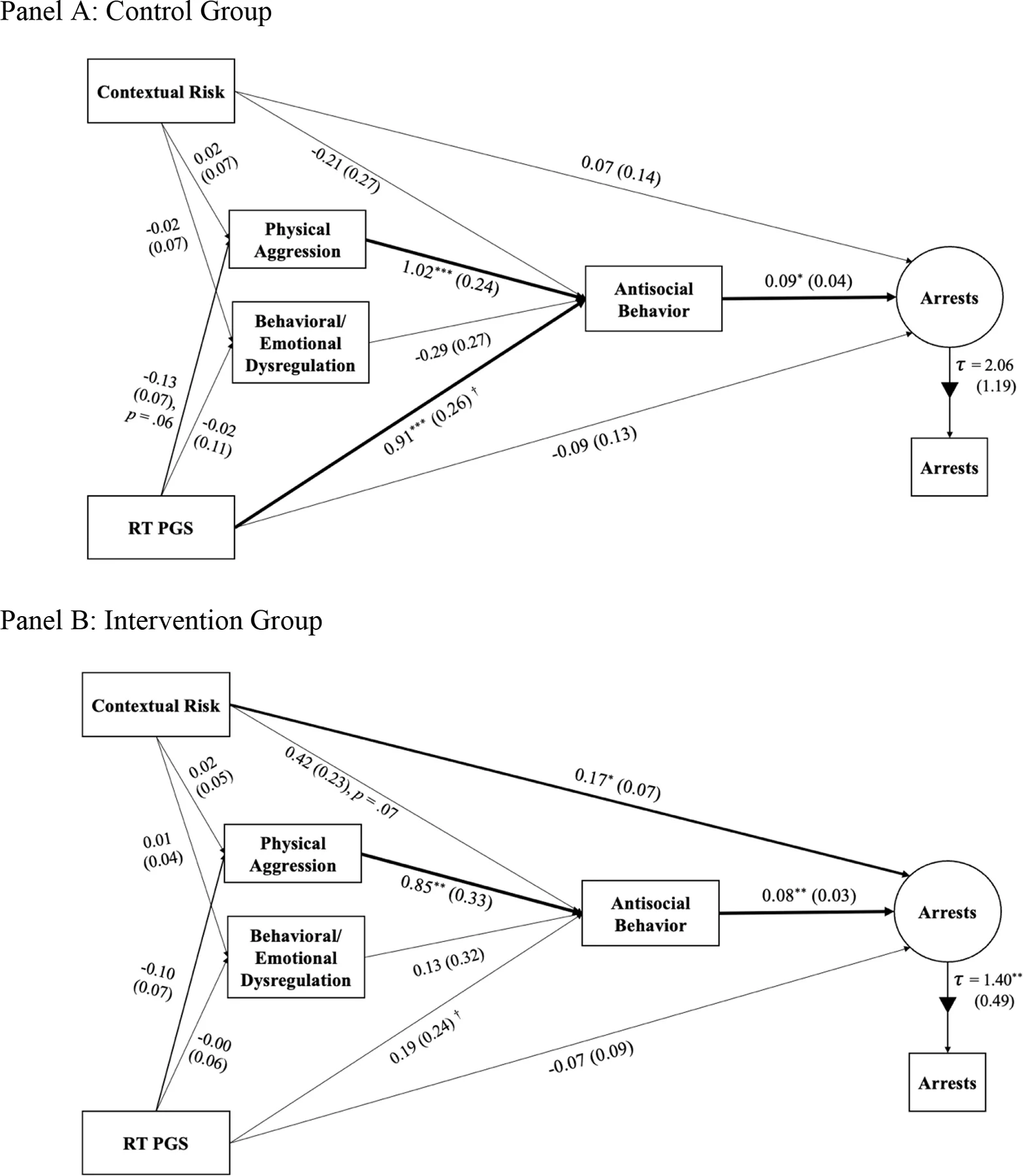
Multigroup Modeling: Similarities and Differences by Intervention Status *Note*. RT PGS = polygenic risk score for risk tolerance. Direct paths and covariates, including sex at birth, age, race, monthly income, and age 2 aggression, were included but not depicted for simplicity. * *p* ≤ .05. ** *p* ≤ .01. *** *p* ≤ .001. ^†^ Represents a significant difference in the effect between groups.

**Table 1.** Frequencies for Criminal Justice Involvement by Race/Ethnicity

1a. Arrests								
	White	Black	Hispanic/Latinx	Asian	NA/NH/PI	Multi-racial	Total Frequency	Valid Percent
Never Arrested	243	167	48	2	23	78	419	78.2
Have Been Arrested	69	49	15	3	13	35	117	21.8
Total	312	216	63	5	36	113	536	100.0
Valid Percent	22.1	22.7	23.8	––	––	31.0		
1b. Incarcerations								
	White	Black	Hispanic/Latinx	Asian	NA/NH/PI	Multi-racial	Total Frequency	Valid Percent
Never Served Time	243	167	48	2	23	78	419	83.0
Served Time	46	41	9	3	11	27	86	17.0
Total	289	208	57	5	34	105	505	100.0
Valid Percent	15.9	19.7	15.7	––	––	25.7		

*Note*. NA = Native American. NH = Native Hawaiian. PI = Pacific Islander. Incarc. = Incarcerated. The valid percent of those incarcerated for Asians and NA/NH/PI were not calculated due to too few individuals represented.

## Data Availability

Behavioral data for ages 7–10 from this study are available from the Child & Family Data Archive (ICPSR 38407, 38745, 38748–49, 38751–55) by following data access procedures. Genetic data are available from dbGaP (phs003442.v1.p1) by following data access procedures. Other study data are available upon reasonable request from the corresponding author.

## Footnotes

1 All findings remained consistent when examining incarceration, rather than arrests, as the outcome.
