# Supplementary material for: Genetic and contextual risks and developmental mechanisms underlying childhood aggression and young adult justice system involvement
Source: Dev Psychopathol. Author manuscript; Available in PMC 2026 Nov 2. (PMC13727081; doi:10.1017/S0954579426101898)
Supplement: Supplementary file 1 [file NIHMS2218108-supplement-Supplementary_Material.docx]

**Supplementary Material**

**Figure S1**

*Conceptual Model of the Developmental Cascade from Childhood Behavioral Aggression and Lack of Control to Trouble with the Law*

*Note.* This is a conceptual model. Childhood aggression, behavioral/emotional dysregulation, and antisocial behavior represent latent factors. Legal trouble indicated whether the child had become involved with the law before the age of 19. Covariates were not depicted for simplicity. The covariates include sex at birth, age, race, and monthly income.

**S1. Stable Trait, Autoregressive Trait, and State (STATE) Models**

Prior to forming latent variables, the stability of the longitudinal predictors (e.g., aggression, dysregulation) across the four time points (i.e., ages 7.5, 8.5, 9.5, and 10.5) were assessed using Stable Trait, Autoregressive Trait, and State (STARTS) models (Donnellan et al., 2012; Kenny & Zautra, 2001). Because the latent variables for childhood behavioral aggression and lack of control would span ages 7.5 to 10.5 across four separate time points––and both behavioral aggression and lack of control were expected to change across middle childhood––the stability of the predictors was assessed to ensure that they were stable enough to prevent the major loss of longitudinal information with the formation of a composite variable. The STARTS model allows for estimation of the extent that the variance of the factor is stable across the developmental time points (e.g., the manifest variables), changing over the time points (e.g., autoregressive traits), or changing moment-to-moment (e.g., state). Utilizing STARTS models ensures the prevention of major loss of longitudinal information with the formation of a composite variable, as the STARTS model allows for estimation of the extent that the variance of the factor is stable across the developmental time points (e.g., the manifest variables), changing over the time points (e.g., autoregressive traits), or changing moment-to-moment (e.g., state). Results indicated that the stable components (i.e., trait and autoregressive latent factors) accounted for the majority of the variance in the repeated assessments across ages 7.5 through 10.5 for both behavioral aggression and behavioral/emotional dysregulation (see Supplementary Material Figures S4-S5). Because the proportion of the variance accounted for by the stable components exceeded that of the random components––ranging from 30.5% to 80.0% for aggression (see Supplementary Material Figure S4) and consistently 99.9% for dysregulation (see Supplementary Material Figure S5), composites for behavioral aggression and dysregulation across middle childhood were formed.

Table S1. Descriptive Statistics and Bivariate Correlations for Primary Study Variables

**Figure S2**

*STARTS Model for Middle Childhood Behavioral Aggression*

*Note*. Model demonstrated acceptable fit: *Δ-2LL* = 5.512; *AIC* = 13777.37; *BIC* = 13813.16; χ^2^(6) = 11.024, *p* = .088; *RMSEA* = .036 [0.0, 0.07], *CFI/TLI* = 0.997, *SRMR* = .024.

**Figure S3**

*STARTS Model for Middle Childhood Behavioral/Emotional Dysregulation*

*Note*. Model fit indices: *Δ-2LL* = 45.00; *AIC* = 9130.35; *BIC* = 9165.66; χ^2^(6) = 90.00, *p* < .001; *RMSEA* = .151 [0.13, 0.18], *CFI/TLI* = 0.785, *SRMR* = .154.

Table S2. Descriptive Statistics and Bivariate Zero-order Correlations for Childhood Behavioral Aggression and Moffitt Dysregulation

|  | | 1 | | | 2 | | | 3 | | | 4 | | | 5 | | | 6 | | | 7 | | | 8 |  |  |
| --- | --- | --- | --- | --- | --- | --- | --- | --- | --- | --- | --- | --- | --- | --- | --- | --- | --- | --- | --- | --- | --- | --- | --- | --- | --- |
| 1. CBCL Aggr. 7.5 | – | | |  | | |  | | |  | | |  | | |  | | |  | | |  | | |  |
| 2. CBCL Aggr. 8.5 | .734^***^ | | | – | | |  | | |  | | |  | | |  | | |  | | |  | | |  |
| 3. CBCL Aggr. 9.5 | .739^***^ | | | .767^***^ | | | – | | |  | | |  | | |  | | |  | | |  | | |  |
| 4. CBCL Aggr. 10.5 | .667^***^ | | | .717^***^ | | | .764^***^ | | | – | | |  | | |  | | |  | | |  | | |  |
| 5. Moff. Dysregulation 7.5 | .240^***^ | | | .167^***^ | | | .177^***^ | | | .121^**^ | | | – | | |  | | |  | | |  | | |  |
| 6. Moff. Dysregulation 8.5 | .206^***^ | | | .146^***^ | | | .183^***^ | | | .118^**^ | | | .560^***^ | | | – | | |  | | |  | | |  |
| 7. Moff. Dysregulation 9.5 | .189^***^ | | | .167^***^ | | | .248^***^ | | | .192^***^ | | | .263^***^ | | | .435^***^ | | | – | | |  | | |  |
| 8. Moff. Dysregulation 10.5 | .198^***^ | | | .160^***^ | | | .216^***^ | | | .226^***^ | | | .328^***^ | | | .401^***^ | | | .354^***^ | | | – | | |  |
| *N* | 567 | | 555 | | | 586 | | | 562 | | | 526 | | | 521 | | | 546 | | | 504 | | | | |
| *M* | 9.46 | | 8.05 | | | 7.90 | | | 7.67 | | | 0.08 | | | 0.06 | | | 0.08 | | | 0.05 | | | | |
| *SD* | 7.16 | | 6.95 | | | 6.79 | | | 6.94 | | | 0.27 | | | 0.23 | | | 0.24 | | | 0.19 | | | | |

*Note*. CBCL = Child Behavior Checklist. Aggr. = Aggression. Moff. Dysregulation = Moffitt Behavioral/Emotional Dysregulation. ^*^ *p* ≤ .05. ^**^ *p* ≤ .01. ^***^ *p* ≤ .001.

Table S3. Descriptive Statistics and Bivariate Zero-order Correlations for Antisocial Behavior and Callous-Unemotional Traits

|  | 1 | 2 | 3 | 4 |
| --- | --- | --- | --- | --- |
| 1. Antisocial Behavior – Age 14 | – |  |  |  |
| 2. CU Traits – Age 14 | .417^***^ | – |  |  |
| 3. Antisocial Behavior – Age 16 | .537^***^ | .286^***^ | – |  |
| 4. CU Traits – Age 16 | .307^***^ | .529^**^ | .422^***^ | – |
| *N* | 553 | 546 | 572 | 566 |
| *M* | 5.77 | 0.71 | 5.54 | 0.66 |
| *SD* | 7.15 | 0.42 | 7.01 | 0.42 |

*Note*. CU = callous-unemotional. ^*^ *p* ≤ .05. ^**^ *p* ≤ .01. ^***^ *p* ≤ .001.
